# Measuring Social Relationships in Different Social Systems: The Construction and Validation of the Evaluation of Social Systems (EVOS) Scale

**DOI:** 10.1371/journal.pone.0133442

**Published:** 2015-07-22

**Authors:** Corina Aguilar-Raab, Dennis Grevenstein, Jochen Schweitzer

**Affiliations:** 1 Institute of Medical Psychology, Center for Psychosocial Medicine, University Hospital Heidelberg, Heidelberg, Germany; 2 Psychological Institute, University of Heidelberg, Heidelberg, Germany; Beijing University of Posts and Telecommunications, CHINA

## Abstract

Social interactions have gained increasing importance, both as an outcome and as a possible mediator in psychotherapy research. Still, there is a lack of adequate measures capturing relational aspects in multi-person settings. We present a new measure to assess relevant dimensions of quality of relationships and collective efficacy regarding interpersonal interactions in diverse personal and professional social systems including couple partnerships, families, and working teams: the EVOS. Theoretical dimensions were derived from theories of systemic family therapy and organizational psychology. The study was divided in three parts: In Study 1 (*N* = 537), a short 9-item scale with two interrelated factors was constructed on the basis of exploratory factor analysis. Quality of relationship and collective efficacy emerged as the most relevant dimensions for the quality of social systems. Study 2 (*N* = 558) confirmed the measurement model using confirmatory factor analysis and established validity with measures of family functioning, life satisfaction, and working team efficacy. Measurement invariance was assessed to ensure that EVOS captures the same latent construct in all social contexts. In Study 3 (*N* = 317), an English language adaptation was developed, which again confirmed the original measurement model. The EVOS is a theory-based, economic, reliable, and valid measure that covers important aspects of social relationships, applicable for different social systems. It is the first instrument of its kind and an important addition to existing measures of social relationships and related outcome measures in therapeutic and other counseling settings involving multiple persons.

## Introduction

Social relationships are an important predictor of health, well-being, and efficacy [1–4]. It follows that human behavior and experiences can be better understood through the interplay and interaction between individuals and contextual factors. Therefore, the evaluation of psychosocial or systemic interventions and their intended process of change raise the question of which measurements and methods should adequately be applied considering the complexity and outcome factors of multiple person therapy and counseling. Existing outcome measures that are generally applied in psychosocial intervention studies focusing on social systems have major disadvantages: Most measures such as the Family Adaptability and Cohesion Evaluation Scale—FACES I-IV [5], the Family Assessment Device—FAD [6] or the Systemic Clinical Outcome and Routine Evaluation—SCORE [7], are customized to specific types of systems such as couples or families. Different or unusual types of social systems such as broader family constellations, cross generational households or arranged teams for short-term trainings and interventions cannot be investigated. Comparisons between different social systems are additionally obstructed by the multitude of constructs, such as family climate, family functioning etc., that are measured. Most of the scales are long and time-consuming in application such as the Systemic Therapy Inventory of Change (STIC) [8], which limits a scale’s utility for both clients and counselors. Some measures focus on more or less stable system characteristics such as family culture. Even though many measures are related to multi-person settings, most only score the individual level [9], based on items addressing the first person singular (“I…”), rather than the perception of the system as a whole (“We…”).

### Theoretical approaches for the understanding of social systems’ interactions and functioning

A social system can be defined as a group of elements, such as individual persons, who are connected to each other by virtue of their relations [10]. Members of social systems interact and communicate in certain ways in order to maintain their dynamic balance and functional abilities [11, 12]. As a self-regulating and autopoietic system they use feedback-loops about their performances based on circular causality [13]. Negative or positive feedback leads either to reducing or enhancing change. Repetitive interactions over time create, in turn, relatively stable structures [14, 15]. The interactive process of informational shift implies that communication always occurs [16–18]. In line with that, interaction and the quality of relational aspects contain more than verbal communication.

The joint interactional reality is ruled by subjective experiences, beliefs, and categories, which may differ strongly between members. Individuals perceive and interpret any kind of stimuli, which in turn gives rise to a certain kind of behavior towards each other [19, 20].

In addition, Bandura’s extended model of perceived *collective efficacy* depicts collective agency and functioning as based on the individuals’ belief in the capability of the group to function as a whole [1]. Unsurprisingly, family efficacy was found to exhibit a substantial influence on the quality of family functioning and satisfaction [21]. The same holds true for organizational contexts [22].

### Systemic intervention techniques and systemic outcome ideals

Despite the heterogeneity of systemic (family) therapy interventions, they all focus on the current problem-maintaining patterns rather than on the etiopathogenetic origins of interactional problems. The target of treatment is not a problematic and symptom-laden individual, but the interactional dynamic between members of a social system. A symptom is always seen as an attempt to a solution and stimulation to further interaction at the same time. Thus, interventions shift from the question of whether any attitude or behavior may be “right” or “wrong”, “normal” or “abnormal”, to whether it has a purpose, beneficial implications, or side effects within the social system. Using techniques such as circular and reflexive questioning [23, 24], the main aims of a therapy are to increase client involvement and participation, and to foster clients’ own unique insight [25]. Based on the guidelines of *not-knowing* what clients should do [26, 27] and *curiosity* about why they do what they do [28], clients are encouraged to find their own useful, unconventional, and uniquely creative way of dealing with problems. The same applies to organizational contexts, where similar systemic techniques are used. Based on this systemic framework, the current study proposes that outcomes should be measured based on the system members’ evaluation of how they get along, rather than their satisfaction with specific outcomes.

### Development of a new scale

Our goal was to create an assessment tool that captures dimensions of the *quality of a relationship* relevant for systemic interventions in interdepended social systems. So far, no measure is applicable to different social systems, such as couple relationships, families, or working teams, and refers to a system in terms of a collective “we”–oneself as part of a certain social system. EVOS tries to fill this gap. The scale was designed to be highly economic to enable easy measurement in therapeutic and counseling settings. As an outcome measure, EVOS was theoretically created to be sensitive to change in order to be used as an evaluation tool for psychosocial or interactional interventions. As outlined above, its development is based on systemic theory and thinking. Multi-perspectivity was taken into account to reflect the systemic-constructivist idea that every person has her own valuable perspective. The aim was to develop a measure without normative presupposition of what constitutes a “good” relationship. The specific content of the items was taken from several models and theories within the field of family research and therapy, as well as from organizational psychology.

EVOS contains two subscales covering affective aspects on one hand with the *quality of relationship* subscale, and cognitive aspects with the *collective efficacy* subscale on the other hand. Items have been derived from different, established models of family therapy as well as models of organizational consulting psychology (c.f. Table 1). Additionally, the EVOS scale includes a consensus item measuring the perceived consensus about the quality of relationships and collective efficacy within the social system. Although the item is not part of the scale, it should enable additional evaluation in terms of the quality of the social systems.

**Table 1:** Item descriptives, factor loadings, and model fit for Study 1.

Items				Theoretical dimensions					EFA		CFA			
Subscale „Quality of relationship”		Cronbach’s α = .82			*M*	*SD*	*P*	*r*_*itc*_	F1	F2	λ C	λ F	λ T	λ O
1. For me, the way we talk with each other, is …				communication	2.21	0.76	73.81	.66	.80	-.05	.68	.80	.70	.54
2. For me, the way we stick together, is …				Cohesion	2.29	0.83	76.23	.64	.56	.21	.71	.82	.79	.65
3. For me, what we do for each other, is …				giving and taking	2.17	0.76	72.44	.57	.44	.25	.61	.66	.69	.50
4. For me, the feeling between us, is …				atmosphere	2.21	0.84	73.62	.71	.88	-.05	.79	.74	.80	.65
Subscale „Collective efficacy“		Cronbach‘s α = .82			*M*	*SD*	*P*	*r*_*itc*_	F1	F2	λ C	λ F	λ T	λ O
5. For me, the way we decide what needs to be done, is. . .				aim	1.90	0.82	63.19	.57	.06	.60	.54	.67	.75	.53
6. For me, the way we recognize what will help us in reaching our goals, is …				ressources	1.91	0.81	63.63	.63	-.05	.75	.68	.71	.74	.57
7. For me, the way we make decisions, is …				decisions	1.89	0.82	62.94	.70	.05	.75	.77	.75	.84	.65
8. For me, the way we find solutions to problems, is …				extending perspective to solutions	1.85	0.82	61.70	.65	.00	.73	.70	.80	.67	.53
9. For me, how we adapt to change, is …				adaptability	1.90	0.83	63.25	.54	.09	.54	.57	.67	.56	.51
total scale Cronbach‘s α = .87					Eigenvalue EFA:				4.55	1.02				
10. I think we will give similar responses to these questions.				consensus item	1.87	0.64								
sample	χ^2^	df	χ^2^ / df	CFI	RMSEA	SRMR			CR	AVE				
Couple (C)	77.188	34	2.270	.928	.078 (.055-.101)	.050			.891	.454				
Family (F)	81.311	34	2.392	.934	.089 (.064-.114)	.044			.919	.535				
Working team (T)	38.952	34	1.146	.991	.031 (.000-.070)	.039			.916	.526				
Overall (O)	121.970	34	3.587	.951	.069 (.056-.083)	.035			.910	.504				

Note: *N*_overall_ = 537, *N*_couple_ = 211, *N*_family_ = 177, *N*_team_ = 149. Answers are given on a four point rating scale, coded from 0 (very poor) to 3 (very good).

Major constructs were established in three influential theories and constitute the theoretical basis of EVOS: In the Beavers Systems Model [29], *family competence* comprises the quality of organizing and managing performances. *Family style* emphasizes the ability of competent families to modify their style to upcoming needs. Important dimensions selected are *structure* of the family, *mythology* describing how the family functions as a group, *goal-directed negotiation*, *and family affect* etc. In the Circumplex Model of Marital and Family Systems, Olson and colleagues discussed three main dimensions: *cohesion*, as a feeling of togetherness towards each other, *flexibility* as a functional ability to change, and *communication* as a facilitating dimension [30, 31]. Lastly, in the McMaster Model of Family Functioning, Epstein and colleagues propose that family functioning is determined by emotional and physical health and problems of family members [32, 33]. The model contains six dimensions: *problem solving*, *communication*, *roles*, *affective responsiveness*, *affective involvement and behavior control*.

In the field of organizational psychology, counseling and team development procedures are of great importance in order to optimize team efficacy. Therefore, team diagnostics are applied assessing the team status to identify what needs to be operationalized and what may be improved [34]. In a coordination network (member-task-tool relation) different component networks are established and investigated, e.g. member-member relationships, or member-task relations [35]. The performance of interacting individuals, groups and superordinate bodies should lay the foundation for *innovations*. Interventions aim at enhancing effectiveness and motivation of task and work processes [36]. The Four Factor Model of facet-specific climate for innovations has been a main focus and functions as a theoretical base for the Team Climate Inventory [37, 38]. It measures four climatic factors: *participatory safety*, *support for innovation*, *vision* and *task orientation*, which can be associated to various stages of the process of group innovations.

### Study Overview

We will describe the development of the EVOS in several steps. Based on a pre-test, Study 1 describes the initial construction of the scale. Study 2 aims to confirm the established measurement model, assesses measurement invariance of the scale in various contexts, and demonstrates construct validity. Finally, an English language translation of the EVOS is evaluated in Study 3.

### Pretest

The first step in the development of the scale included the formulation of items based on the theoretical background presented in the introduction. Twenty-four experts working more than 10 years in the field of systemic research and therapy participated in the process. This resulted in a pool of 30 items, which was tested in a small sample of *N* = 31 participants, both in private (*n* = 16) as well as in organizational (*n* = 15) settings. Participants were asked to comment on various aspects of the questionnaire, such as comprehensibility and clarity of the items. Two different response scale formats were considered, a visual analog scale marked from 0% to 100% and a 4-point rating scale. Participants’ feedback was overall positive and indicated a preference for the 4-point rating scale format ranging from “very poor” (0) to “very good” (3). To get a first impression of test-retest reliability, participants completed the same questionnaire after an interval of three weeks. Test-retest correlations amounted to *r* = .66 for quality of the relationship and *r* = .56 for the collective efficacy subscale. This indicated a moderate stability. Following the pre-test, for each item representing one dimension at least one additional alternative item wording were generated without altering the related theoretical dimensions. This resulted in a pool of 80-items that was subsequently tested in a large sample.

## Study 1: Construction of the EVOS

### Methods

#### Participants and Procedure

Participants for the first study were recruited online and by means of a paper-pencil-survey, at the University of Heidelberg and in different local training institutions, via local newspapers as well as private and public networks. There was a clarification of objectives of the study, voluntary participation and the possibility of cancellation at any time. Participants did not provide written formal consent as this study was in part conducted online. They were informed that by returning the questionnaires they would consent to participation in the study and their data being analyzed. The study including the procedure was approved by the ethics committee of the Heidelberg Medical Faculty (S-508/2012). Each participant was free to choose which social context (partnership, family, working team) to evaluate. A total of 546 individuals took part in the study. Nine participants were excluded from the data analysis due a large amount of missing data (>10%), resulting in *N* = 537 participants (41% pen-paper-version, 59% online; *n* = 402 (74.9%) female and *n* = 131 (24.6%) male). The average age of the participants was 32 (*M* = 32.00, *SD* = 13.04). The majority of the participants, *n* = 253 (47.5%), were school and university students or apprentices, *n* = 244 (45.8%) were employed persons and *n* = 36 (6.7%) stated to be unemployed or “other”. Concerning the possible target contexts of the EVOS, *n* = 211 (39.3%) evaluated their couple relationship, *n* = 177 (33.0%) their family relationship (ranging from two to ten family members), and *n* = 149 (27.7%) rated their working teams (ranging from two to sixty team members). A full information maximum likelihood estimator was used to handle missing data during parameter estimation (a total of 0.4% of all cells).

#### Statistical Analysis

SPSS 21 [39] was used for descriptive and exploratory factor analyses (EFA), and M*plus* 7.11 [40] was used for confirmatory factor analyses (CFA). For all CFAs, a maximum likelihood estimator with robust standard errors (MLR) was used. M*plus* provides MLR for maximum likelihood with robust ‘Huber-White’ standard errors and a scaled test statistic asymptotically equivalent to the Yuan–Bentler T2* statistic [41, 42]. Robust procedures were required due to the 4-point rating scale and because Small’s omnibus test indicated that multivariate normality did not hold in our sample, χ^2^ (22) = 200.74, *p* < .0001. Model fit was evaluated by 1. the—ideally non-significant—χ^2^ test [43] and as low as possible a χ^2^/*df* ratio, ideally as low as 2 [44]; 2. the comparative fit index (CFI) with values of .90/.95 and above indicating appropriate/good model fit [45, 46]; 3. the root mean square error of approximation (RMSEA) with values of .05/.08 and less indicating good/reasonable model fit [47]; and 4. the standardized root mean square residual (SRMR) with values less than .08 considered to reflect good fit [46]. For comparisons of nested models later on, the Bayesian Information Criterion (BIC) was used with smaller values indicating better model fit [48].

### Results

Besides computing item difficulties, corrected item-to-total correlations and internal consistencies for the two sub-scales and the total scale, we conducted an exploratory factor analysis (EFA; principal axis factoring) as shown in Table 1.

In order to select items from the large 80 items pool and to extract the minimum amount of factors we used Promax rotation to allow for correlated factors. To test the sufficiency of the sample and data quality [49], we calculated the Kaiser-Meyer-Olkin criterion (.90) as well as Bartlett’s test of sphericity, χ^2^ (36) = 1956,154, *p* < .001. Both tests indicated sample adequacy and the necessary substantial correlations to conduct a factor analysis. After a successive process, each time deleting items with loadings lower than .40, lastly the Kaiser-Criterion (eigenvalue EV > 1) resulted in a two-factor solution with nine items in line with the theoretical framework: The first factor accounted for 50.5% of the variance and the second factor for 11.3%. Inter-item correlations can be seen in Table 2.

**Table 2:** Inter-item-correlations for the EVOS for Study 1 (above diagonal) and Study 2 (below diagonal).

	COM	COH	G&T	ATM	AIM	RES	DEC	SOL	ADA
communication	-	.51	.44	.68	.35	.34	.42	.39	.30
cohesion	.60	-	.52	.57	.36	.40	.47	.40	.45
giving and taking	.53	.68	-	.49	.39	.39	.44	.39	.34
atmosphere	.66	.66	.62	-	.39	.39	.48	.41	.39
aims	.45	.51	.50	.49	-	.46	.54	.43	.37
resources	.49	.49	.53	.53	.61	-	.55	.56	.39
decisions	.50	.49	.49	.51	.60	.61	-	.56	.49
solutions	.53	.54	.52	.59	.53	.62	.67	-	.46
adaptability	.48	.50	.48	.54	.51	.53	.55	.63	-

Note: All correlations are significant at *p* < .001.

As expected, the difficulty indices were mediocre, but generally >.50. All items were "easy" to rate, so that both the relationship quality and effectiveness were mainly judged as positive–as would be expected in a non-clinical sample. The corrected item-to-total correlations with no item lower than .50 were consistently high, ensuring a high level of representativeness of the subscales. The factor analysis yielded two factors (subscales) on which the items loaded at least .40 with cross-loadings lower than .25. The internal consistencies can be judged as moderate, which is due to the short scale-length and weak to moderate inter-item-correlations (as an indicator for homogeneity).

To evaluate the accuracy of the two-factor structure of the 9 items obtained by EFA (the consensus item is not an integral part of the scale), a CFA was conducted separately for each social system (couple, family and working team) as well as overall across all social contexts. This was done for the reader’s convenience only and will be reevaluated in Study 2. Model fit was acceptable for all CFAs, while the scale showed best fit in a working team context and worst fit in the couple context Table 1. Additionally, the CFA enabled us to overcome the Alpha coefficients inadequacy to estimate reliability of a scale that lacks true tau-equivalence and strict uni-dimensionality. SEM based estimates can be used instead [50, 51]. A measure is considered to be reliable if at least half the variance can be extracted by the construct, AVE (average variance explained) > .50, and if the construct reliability (CR or Ω_w_) passes a threshold of .60. The EVOS met both criteria.

## Study 2: Validation

With Study 2, we aimed to replicate the previously established measurement model for the EVOS. Additionally, we investigated measurement invariance between different contexts to demonstrate that EVOS assesses the same construct of social relationship, even in noticeably dissimilar situations. In a last step, we investigated correlations with other relevant measures to validate the EVOS.

### Methods

#### Participants and Procedure

Sample 2 included *N* = 564 participants, of whom six were excluded from the data analysis due to >10% of their data missing. This resulted in *N* = 558 participants (45.5% pen-and-paper-version, 54.5% online; *n* = 426 females, *n* = 132 males). Again, participants where free to choose which social context to evaluate, after being informed about the objectives of the study, the voluntariness and cancellation option. Participants were informed that by returning the questionnaires they would consent to participation in the study and their data being analyzed. Participants of this study did not provide written formal consent as this study was in part conducted online. The study including the procedure was approved by the ethics committee of the Heidelberg Medical Faculty (S-508/2012). Only participants in the pen-paper condition received additional questionnaires due to copyright reasons. The average age was 33 (*M* = 33.00; *SD* = 12.31). Sample composition was comparable to Study 1 with the majority of the participants being students (*n* = 266; 40.6%), followed by employed persons (*n* = 142; 25.5%) and unemployed or “else” (*n* = 189; 33.9%). Evaluation targets were balanced, with *n* = 182 (32.6%) participants evaluating their couple relationship, *n* = 188 (33.7%) evaluating their families and the same number of *n* = 188 rating their working teams. A total of 1.14% of all data were missing at random (MCAR) and subsequently handled using a full information maximum likelihood estimator. Again, robust procedures were required for the CFA, because Small’s omnibus test indicated that multivariate normality did not, χ^2^ (22) = 210.06, *p* < .001.

#### Statistical Analysis

Measurement invariance (MI) refers to the measurement model being invariant across groups, settings, or times of measurement [52, 53]. In the case of EVOS, one crucial aspect is the applicability of the scale in different contexts. Testing for MI can provide a statistical assessment of EVOS’ ability to capture social relationships across a broad range of contexts. Testing for MI involves a series of tests using consecutive multi-group CFAs (MGCFA) with increasingly restrictive nested models. Model parameters are set equal across groups and model fit is evaluated to examine whether a more restrictive model can still similarly represent the empirical data. If invariance holds at a given level, fixing the relevant parameters to be equal across groups should not harm model fit.

Testing for MI includes several hierarchically ordered levels. At the first level of *configurable invariance*, a similar but not necessarily the same construct is measured in both groups. If the model holds, all items are associated with the same latent variable(s), though the factor loadings can differ across groups. A failure to replicate the two-factor model of the EVOS would indicate a severe difference across contexts. This step differs from prior analyses by using MGCFA, rather than single group CFA. At the level of *metric invariance* the factor loadings are set equal across groups. This demonstrates that respondents attribute the same meaning to the items. Therefore, the underlying latent construct is assumed to be identical. If metric invariance holds, relationships to other measures, i.e. correlations, can be meaningfully interpreted. This is a crucial test, as it could very well be possible that respondents attribute different meaning to the items in different contexts. For example, the item “For me, the way we talk with each other, is …” could be construed differently in a working team context (i.e., being polite and friendly) than in a couple relationship context (i.e., telling your spouse that you love him/her). The next level of *scalar invariance* means that items are calibrated equally in different contexts. Statistically, item intercepts are held equal across groups. Only then can we assume that scores not only have the same unit of measurement, but also the same origin in the regression equations underlying the MGCFA. If scalar invariance holds, different social contexts can be compared regarding their scores on the latent variable. Lack of scalar invariance could be due to some items being easier or more difficult to endorse in one context than another. With regard to the theoretical conception of the EVOS, it is highly unlikely that this level of invariance could hold. Based on this, any further steps in testing for MI are not expected to succeed, so we will not go into any more details.

When testing for MI, subsequent models can at each step be compared using an ideally non-significant χ² difference test. This test, however, tends to be too strict as sample size increases, so model fit indices can be used alternatively to assess model fit. Conventionally, a CFI drop is accepted if smaller than .01 and a RMSEA increase would still be acceptable if smaller than .015 [54, 55]. Additionally, BIC indicates at each step whether the model remains as accurate as before, representing a tradeoff between accuracy and parsimony.

#### Measures

Even though EVOS is applicable to different contexts, validation measures are more often than not specific. Thus, participants in the pen-paper-condition were presented different measures depending on the context they chose to evaluate.

**Satisfaction with life scale (FLZ):** The FLZ (“Fragebogen zur Lebenszufriedenheit”; [56]) is a commonly used German language measure of satisfaction with several aspects of life. The measure includes ten sub-scales with seven items each referring to 1. Health, 2. Work and job, 3. Financial situation, 4. Leisure, 5. Marital and couple relationship, 6. Relationship to one’s own children, 7. Own person, 8. Sexuality, 9. Friends and relatives, and 10. Living situation. Answers are given on 7-point scales marked from 1 (very dissatisfied) to 7 (very satisfied). Cronbach’s Alpha for the subscales ranged from .73 to .89 (FLZ_tot α = .95) in our sample. Sum scores can be computed for individual scales. Additionally, a sum score for global life satisfaction includes the subscales health, financial situation, leisure, own person, sexuality, friends and relatives, and living situation. To validate the EVOS, only those subscales which were applicable in a context were used.

**Family scales (FB):** The family scales ("Familienbögen" [57] are a self-evaluation measure to assess family functioning and resources. It is based on a general process model of family functioning [58] and includes seven sub-dimensions: task fulfillment, role behavior, communication, emotionality, affective responsiveness, control, and values/rules. Different versions of the FB are tailored towards specific contexts. The FB-A (or FB-fam) targets whole family systems, whereas the FB-Z (or FB-two) focuses on dyadic relationships.

The FB-two consists of 28 items in seven sub-scales. The FB-fam goes beyond the FB-two by additionally including 12 more items measuring social desirability and defensive mechanisms. Answers are given on four-point scales marked from 1 (completely true) to 4 (not true at all). All 28 items of the seven core scales can be summed up to global score of functioning. Cronbach’s Alpha amounted to α = .87 for FB-two and α = .74 for FB-fam. The FB scales have been shown to be responsive to therapeutic interventions [59] and resemble a global assessment of family relationships, as it is covered by the EVOS.

**Work in a team scale (FAT):** The work in a team scale ("Fragebogen zur Arbeit im Team"; [60]) is based on two models of team development, the SGRPI-Modell [61] and the theory of team reflexivity [62, 63]. The FAT includes 24 double-items. Answers are given on 6-point scales with one end marked with a positive sentence (e.g., “goals in our team are clear to us”) and the other end marked with a corresponding negative statement (e.g., “goals in our team are not clear to us”). Items are grouped in four factors: goal orientation, task achievement, coherence, and responsibility. A sum score can be computed over all items with lower scores proposing to indicate a need for a team development intervention. In our sample Cronbach’s Alpha reached α = .91 for the scale sum score.

### Results

#### Confirming the measurement model for the EVOS

Inter-item correlations Table 2 were slightly higher than in the first study, varying in a medium range. The measurement model that was established previously was now subject to confirmation. Table 3 shows the results for the psychometric evaluation of the EVOS. Similar to Study 1, the item difficulty coefficients can be judged as mild, still all >.50, indicating a positive evaluation of the respective social contexts, overall. The high corrected item-to-total correlations (all >.50) demonstrated good representativeness for the subscales. Internal consistencies were satisfactory to very good. A model with two interrelated factors fitted the empirical data well across all contexts. Again, model fit was slightly better in the working team context than in the significant other context.

**Table 3:** Item descriptives, factor loadings, and model fit for Study 2.

Items				Theoretical dimensions					CFA			
Subscale „Quality of relationship“		Cronbach‘s α = .87			*M*	*SD*	*P*	*r*_*itc*_	λ C	λ F	λ T	λ O
1. For me, the way we talk with each other, is …				communication	2.09	0.78	69.71	.68	.80	.72	.78	.75
2. For me, the way we stick together, is …				cohesion	2.27	0.83	75.57	.75	.74	.82	.85	.81
3. For me, what we do for each other, is …				giving and taking	2.24	0.78	74.73	.67	.68	.78	.76	.77
4. For me, the feeling between us, is …				atmosphere	2.11	0.83	70.25	.76	.78	.86	.82	.83
Subscale „Collective efficacy“		Cronbach‘s α = .88			*M*	*SD*	*P*	r_itc_	λ C	λ F	λ T	λ O
5. For me, the way we decide what needs to be done, is. . .				aim	1.90	0.82	61.05	.67	.66	.67	.79	.72
6. For me, the way we recognize what will help us in reaching our goals, is …				ressources	1.91	0.81	59.68	.72	.73	.77	.77	.77
7. For me, the way we make decisions, is …				decisions	1.89	0.82	63.20	.73	.78	.81	.78	.79
8. For me, the way we find solutions to problems, is …				extending perspective to solutions	1.85	0.82	63.20	.74	.82	.82	.81	.82
9. For me, how we adapt to change, is …				adaptability	1.90	0.83	64.82	.67	.67	.73	.72	.73
total scale Cronbach‘s α = .92												
10. I think we will give similar responses to these questions.				Consensus item	1.99	0.69						
sample	χ^2^	df	χ^2^ / df	CFI	RMSEA	SRMR		CR			AVE	
Couple (C)	46.639	26	1.794	.967	.066 (.034-.096)	.039		.916			.550	
Family (F)	38.095	26	1.465	.985	.050 (.000-.082)	.030		.932			.605	
Working team (T)	36.186	26	1.391	.988	.046 (.000-.079)	.028		.936			.620	
Overall (O)	69.616	26	2.678	.981	.055 (.039-.071)	.023		.932			.605	

Note: *N*_overall_ = 558, *N*_couple_ = 182, *N*_family_ = 188, *N*_team_ = 188.

#### Measurement invariance

Results of the MI tests can be seen in Table 4. In agreement with our expectations, metric invariance holds across all contexts. Further restrictions, required by higher levels of invariance, severely diminished the model fit and could not be accepted. Our results confirm the structural validity of the EVOS. The previously developed measurement model could be replicated. Tests of measurement invariance indicated that EVOS indeed measures the same latent construct in all contexts.

**Table 4:** Measurement invariance between contexts couple, family, and working team.

MGCFA comparison		equal loadings	equal intercepts	equal residuals	equal means	*df*	χ^2^	Δ*df*	Δχ^2^	*p*	CFI	RMSEA	BIC
										(n.s.)	(> .95)	(< .05)	(lower)
1	configural invariance	-	-	-	-	78	120.26***	-	-	-	.981	.054	9872
**2**	**Weak/metric invariance**	**X**	**-**	**-**	**-**	**92**	**149.41** **	**14**	**30.84**	**.006**	**.974**	**.058**	**9813**
3	strong/scalar invariance	X	X	-	-	106	228.83***	14	88.77	< .001	.945	.079	9812
4	strict invariance	X	X	X	-	124	259.45***	18	30.01	.037	.939	.077	9730
5	mean invariance	X	X	-	X	110	273.24***	4	47.52	< .001	.927	.090	9837
6	full invariance	X	X	X	X	128	304.92***	4	47.68	< .001	.921	.086	9755

Note: *N*_overall_ = 558, *N*_couple_ = 182, *N*_family_ = 188, *N*_team_ = 188. Values in parentheses refer to criteria for good model fit.

*** *p* < .001

** *p* < .01.

The accepted model is written in bold.

#### Validation

Descriptives as well as correlations between the EVOS and the validation measures can be seen in Table 5. In the couple context, EVOS showed the expected high and positive correlations with scales of life satisfaction, especially with the subscale of marriage/partnership, whereas lower correlations with the subscale sexuality are plausible due to limited content overlap. Based on a negative or problem-oriented approach within the family scales–dyadic relationships version–substantial negative correlations have been identified, confirming our hypothesis. For the evaluation of the family context similar moderate to very strong results indicated high construct validity. In the working team context slightly lower correlations were found. Still, all correlations pointed in the expected direction. Overall, the highest construct overlap could be found for private social systems, especially with regard to the total score of the life satisfaction scale, which does not include all subscales of the FLZ. To summarize, EVOS seems to measure different aspects of social relationships, but still showed an adequate overlap with existing measures. Thus, EVOS demonstrated convergent, as well as discriminant validity.

**Table 5:** Descriptives and Pearson correlations with EVOS for validation measures in Study 2.

	couple				family				team			
	*n*	*M*	*SD*	*r*	*n*	*M*	*SD*	*r*	*n*	*M*	*SD*	*r*
EVOS (total)	182	20.27	4.88	-	188	17.38	5.95	-	188	16.58	5.77	-
FLZ (total)	75	259.32	29.78	.60**	84	255.52	28.56	.45**	95	252.45	32.06	.30**
FLZ (marriage/partnership)	74	41.91	5.73	.72**	44	37.73	7.45	.49**	-	-	-	-
FLZ (sexuality)	75	38.2	6.13	.31**	-	-	-	-	-	-	-	-
FLZ (relationship to children)	-	-	-	-	28	39.89	8.15	.47*	-	-	-	-
FLZ (job)	-	-	-	-	-	-	-	-	94	34.4	7.31	.54**
FLZ (financial)	-	-	-	-	-	-	-	-	95	34.53	7.4	.25*
FB-Z (two/partnership)	75	17.77	9.81	–.66**	-	-	-	-	-	-	-	-
FB-A (family)	-	-	-	-	84	25.94	14.49	–.83**	-	-	-	-
FAT (total)	-	-	-	-	-	-	-	-	95	89.13	19.06	.72**

** *p* < .01

* *p* < .05

## Study 3: The English Version of the EVOS

### Methods

#### Participants and Procedure

The EVOS was translated into English and back translated with the support of two native speakers. Afterwards, the recruitment took place in England in three centers of KIDS Company in London (http://www.kidsco.org.uk/), an institution that works with children and adolescents applying systemic interventions [64]. All participants were informed about the study objectives, the voluntariness and the possibility to drop out at any time. The recruitment took place in a staff member meeting. After formal written consent *N* = 330 employees of KIDS Company participated. Data from *n* = 13 were excluded as a result of more than 10% of their data missing. The validation study included *n* = 317 participants (239 female, 75.4%; 77 male, 24.3%; one undetermined, 0.3%) with a mean age of 36.07 years (*SD* = 10.56). Participants were free to choose whether they wanted to evaluate their couple relationship (*n* = 90), family (*n* = 112), or working team (*n* = 115). A total of 1.2% of all data were missing (at random) and subsequently handled using full information ML in the CFA.

### Results

#### Descriptive Data Analysis

Comparable to the German participants in the earlier studies, the UK participants also rated their couple relationships more favorably than their family relationships and working team relationships, respectively, with regard to the EVOS means, *F*(2, 314) = 8.83, *p* < .001, η^2^ = .05, *M*s = 2.31, 2.06, 1.98, *SD*s = 0.06, 0.05, 0.05. Both subscales of the EVOS reflected this tendency; quality of the relationship: *M*s = 2.47, 2.12, 2.12, *SD*s = 0.06, 0.06, 0.06; collective efficacy: *M*s = 2.19, 1.95, 1.88, *SD*s = 0.07, 0.06, 0.06. This also implies that participants’ ratings, across all contexts, were higher for quality of the relationship than for collective efficacy, *t* = 10.48, *df* = 316, *p* < .001, Cohen’s *d* = 1.18. Cronbach’s Alpha for the scale amounted to α = .93. Item to total correlations ranged from *r*_*itc*_ = .70 to .78.

#### Confirmatory Factor Analysis

**Fig 1:**
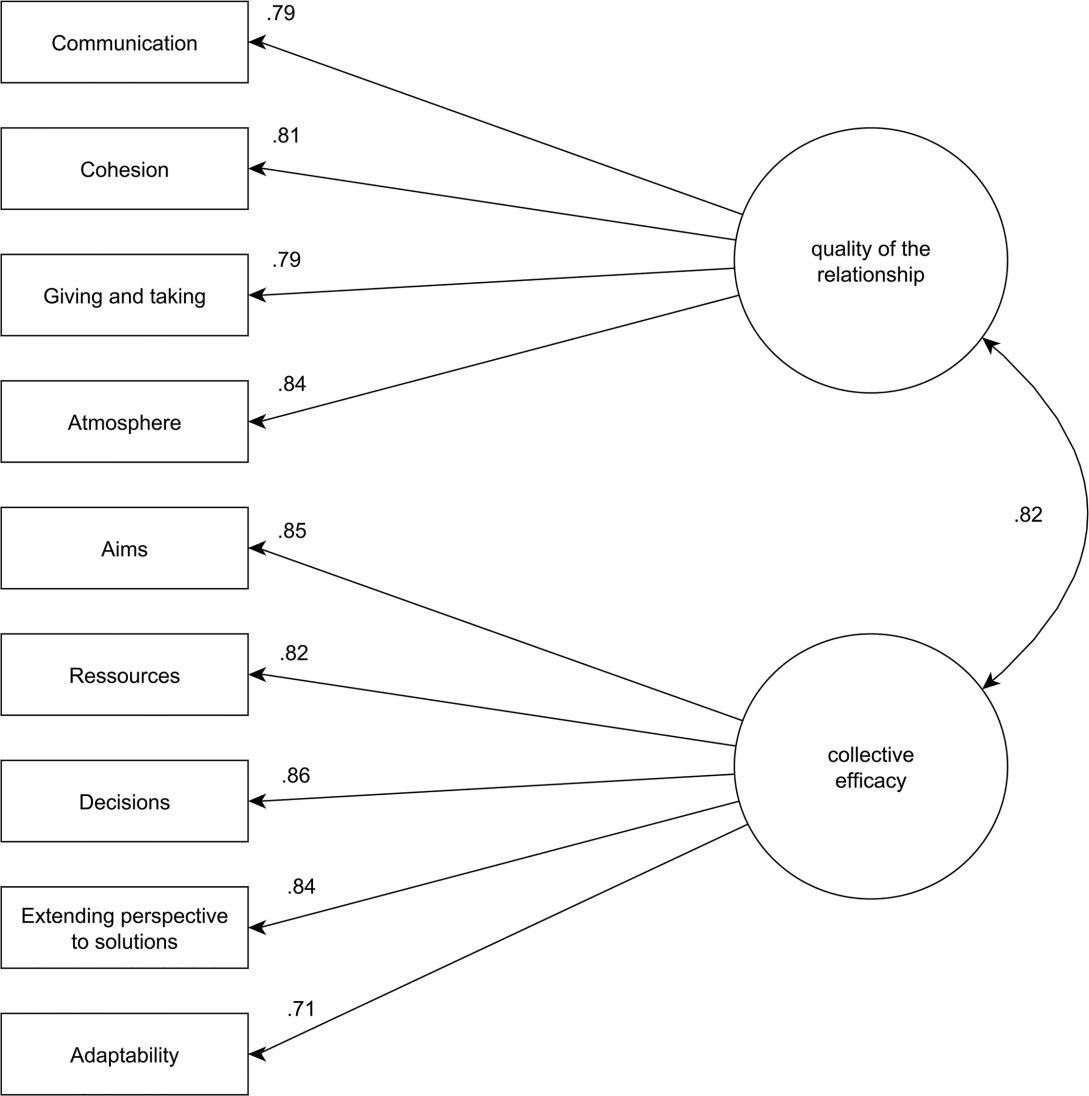
EVOS factor model (Study 3) depicting standardized factor loadings.

We aimed to replicate the initially presented two-factor model for the English language translation of the EVOS. The model fitted the data well, χ^2^(26) = 54.36, χ^2^/*df* = 2.09, *p* < .001, RMSEA = .063, CFI = .977, SRMR = .030. Fig 1 shows standardized loadings for this model. The construct reliability yielded Ω_w_ = .95 while the AVE amounted to .66. To summarize, the English translation of the EVOS was successfully tested. The measurement model that was developed for the original German version also held for the English adaptation.

## Discussion

The aim of the present research was to construct and validate a short scale to measure *quality of relationships* and the *collective efficacy* in different social systems as systemic interventions are similarly applied in different social contexts. Theories of functional social systems with regard to the context of couple/family therapy and organizational psychology, as well as systemic interventional guidelines provided the theoretical framework based on systemic, non-normative and constructivist approaches and referring to important theories of family functioning, family style as well as organizational theories of working teams. Study 1 presented the initial construction of the scale, resulting in a 9 plus 1 item two-factor solution. Study 2 confirmed the good psychometric properties of the EVOS and the established measurement model. Moreover, we were able to confirm metric measurement invariance across different social contexts, confirming the applicability in many different contexts. Construct validity of the scale was demonstrated by correlations with measures of life satisfaction, family functioning, and team assessment. In Study 3 we validated an English translation of the EVOS, which also demonstrated good psychometric qualities and confirmed the original measurement model.

With EVOS, we present a short, economic, reliable, and valid measure answering a need for a measure taking into account affective and cognitive aspects of social systems relationships. Despite high correlations with corresponding measures that assess specific aspects of functioning or pathology, EVOS covers theoretically and directly the core concept of change behind systemic interventions, focusing on relational and dynamic aspects that are important in different social systems.

Within the theoretical framework of systemic counseling, the diagnosed ‘problem’ cannot be understood as an objectified ‘true psychopathology’. The primary concerns of systemic interventions are to address the requests of all members of a social system, to ensure task clarification, and to critically question the functionality of symptoms, in order to contextualize them within the interactional, reciprocal framework. This should ultimately initiate a change with no predictable linear outcome. The evaluation of such interventions becomes even more difficult when the intended goal is highly individual or when the fluctuation of the ‘problem definition’ is taken into account. For example, the problem of a non-eating child at the beginning of therapy might be replaced by the problem of parents not talking to each other anymore during the progress of the therapy process. A measure, which directly addresses crucial aspects of social relationships, could reveal such structures important for the counseling process. Still, the changeable nature of initial problems contains several problems in evaluating interventions that can hardly be overcome by quantitative measures.

### Directions for Future Research and Limitations

Our long-term goal was to develop a scale capable of measuring the change following interventions addressing relational aspects. As we conducted primarily cross-sectional studies, we are unable to evaluate EVOS ability to measure change. This highly important issue will be dealt with in future longitudinal studies. Moreover, in almost all of the studies presented here, we got an unintended unbalanced composition of the samples with respect to gender. Future studies will need to focus on verification of invariance across genders in order to take into account that relationship perception and satisfaction might vary across genders [65–67]. Further on, all participants answered the measures with regard to only one social context due to practical reasons. Hence, the subsamples evaluating different contexts (partnership, family, working team) include different individuals and therefore might differ from each other.

Unsurprisingly, the sole use of self-report measures has essential limitations [68]. In order to broaden the perspective of the evaluation of one particular social system and to investigate the fit between different perspectives referring to second order cybernetics, a parallel external rating version for counselors and therapists (EVOS-E) was also created, but is still subject to evaluation. Furthermore, it is yet to be established whether EVOS can be applied to children between the age of 12 and 18, as they are important family members giving genuine insights of a family’s system quality. Moreover, the application in a clinical sample is necessary to identify its potential to detect the correspondence between pathology and negative evaluation of the quality of social relationships.

Simple questionnaires can never fully meet the requirements concerning the complexity of social systems. Nonetheless, they can still form the basis for many complex methodologies such as time series panel analysis [69] or multilevel growth modeling [70]. To get a more comprehensive view of social systems and their characteristic hierarchical data sets, we need to investigate different approaches that go beyond sum scores, means, and difference values [71]. The correlations between members of a social system can be of special insight for the intervention process. Therefore, the EVOS scale includes one additional item assessing the perceived consensus in a system. The differences between actual and expected consensus of the evaluation by each member could potentially illuminate essential aspects of a social system’s dysfunctional or problematic relationship patterns [72–74]. This might provide valuable new insights for therapy processes and outcome research.

### Conclusion

EVOS is a reliable, valid and economic tool measuring the perceived *quality of relationship* and *collective efficacy* in personal and organizational social systems. We see it as an important addition to fill the gap in current diagnostic research particularly with regard to systemic and other relational psychosocial interventions.

## Supporting Information

S1 File EVOS Scale English Version. (PDF)

S2 File EVOS Scale German Version. (PDF)
