# Supplementary material for: Measuring Social Relationships in Different Social Systems: The Construction and Validation of the Evaluation of Social Systems (EVOS) Scale
Source: PLoS One. 2015 Jul 22;10(7):e0133442. doi: 10.1371/journal.pone.0133442 (PMC4511583; doi:10.1371/journal.pone.0133442)
Supplement: S1 File — (PDF) [file pone.0133442.s001.pdf]

In EVOS you can assess **one** of your important social systems (partnership, family, work team etc.). EVOS is applicable, whenever two or more people affiliated with each other, when they live or work together.

Please choose one of your following social systems you are belonging to for answering this questionnaire:

…your partnership ☐

…your family ☐ ____(number of members)

…your work team ☐ ____(number of members)

…other ☐ ____(number of members)

You are member of this social system (partnership, family, work team or other)

since: _________________ (e.g. March 2012)

Please refer to the **last two weeks** when answering the questions. For every statement, mark the answer that comes closest to your experience. If in doubt, follow your first impulse. Please take a moment at the end to **make sure you have ticked a box in every line**.

	**very poor**	**poor**	**good**	**very good**
1. For me, the way we talk with each other, is …				
2. For me, the way we stick together, is …				
3. For me, what we do for each other, is …				
4. For me, the feeling between us, is …				
5. For me, the way we decide what needs to be done, is …				
6. For me, the way we recognize what will help us in reaching our goals, is …				
7. For me, the way we make decisions, is …				
8. For me, the way we find solutions to problems, is …				
9. For me, how we adapt to change, is …				
10. I think we will give similar responses to these questions.	**Strongly disagree** ☐	**Disagree** ☐	**Agree** ☐	**Strongly agree** ☐
