# Supplementary material for: Measuring Social Relationships in Different Social Systems: The Construction and Validation of the Evaluation of Social Systems (EVOS) Scale
Source: PLoS One. 2015 Jul 22;10(7):e0133442. doi: 10.1371/journal.pone.0133442 (PMC4511583; doi:10.1371/journal.pone.0133442)
Supplement: S2 File — (PDF) [file pone.0133442.s002.pdf]

In EVOS können Sie eines Ihrer für Sie wichtigen **sozialen Systeme** (Paarbeziehung, Familie oder Arbeitsteam o.a.) einschätzen. Er ist überall anwendbar, wo zwei oder mehr Menschen miteinander verbunden sind, zusammen leben oder arbeiten.

Bitte entscheiden Sie sich beim Beantworten für eines Ihrer folgenden sozialen Systeme:

…Ihre Paarbeziehung ☐

…Ihre Familie ☐ ____________ (Mitgliederzahl)

…Ihr Arbeitsteam ☐ ____________ (Mitgliederzahl)

…Sonstiges/Welches _______________________ ☐ ____________ (Mitgliederzahl)

Sie sind Mitglied dieses Systems (Paarbeziehung, Familie, Arbeitsteam o.a.)
seit: ________________ (z.B. März 2012)

Bitte denken Sie bei jeder Aussage an **nur eines Ihrer sozialen Systeme** (entweder Ihre Paarbeziehung, Ihre Familie oder Ihr Arbeitsteam o.a.) in den **letzten 14 Tagen**. Bitte beantworten Sie **alle** Aussagen mit **einem** Kreuz, selbst wenn Sie sich Ihrer Einschätzung nicht völlig sicher sind.

	**nicht gut**	**eher nicht gut**	**eher gut**	**gut**
1. Wie wir miteinander reden, finde ich …				
2. Unseren Zusammenhalt finde ich …				
3. Was wir füreinander tun, finde ich …				
4. Die Stimmung unter uns finde ich …				
5. Wie wir verabreden, was getan werden soll, finde ich…				
6. Wie wir erkennen, was uns beim Erreichen unserer Ziele hilft, finde ich …				
7. Wie wir Entscheidungen treffen, finde ich …				
8. Wie wir neue Lösungswege finden, finde ich …				
9. Wie wir uns auf Veränderungen einstellen, finde ich …				
10. Ich denke, dass wir (die Systemmitglieder) diese Fragen ähnlich beantworten.	**stimmt nicht** ☐	**stimmt eher nicht** ☐	**stimmt eher** ☐	**stimmt genau** ☐
